# Metal-Based Complexes in Cancer

**DOI:** 10.3390/ijms24087289

**Published:** 2023-04-14

**Authors:** Claudia Riccardi, Marialuisa Piccolo

**Affiliations:** 1 Department of Chemical Sciences, University of Naples Federico II, 80126 Naples, Italy; 2 Department of Pharmacy, School of Medicine, University of Naples Federico II, 80131 Naples, Italy

Metal-based drugs have attracted growing interest in biomedicine [1] due to their potential value for both therapeutic and diagnostic applications in different diseases, especially in cancer [2,3,4], with several examples of compounds recently reaching preclinical or advanced clinical evaluation [5,6,7,8,9,10,11,12].

Indeed, metal-containing complexes are endowed with impressive chemical diversity and versatility, depending on the metal of choice, its oxidation state, the number and type of coordinating ligands, and specific magnetic and/or optical properties.

This Special Issue aimed to highlight the most recent advances in metal-based complexes used in cancer therapy and diagnostics.

The interest in metal-based drugs started in the early 1960s with the serendipitous discovery of the first platinating agent, i.e., cisplatin, which was approved in the 1970s by the Food and Drug Administration (FDA) for the treatment of many solid tumours, including testicular, ovarian, bladder and colorectal cancers. Following cisplatin, several platinum-containing derivatives were developed, although only two were globally approved for usage in the clinic, which are carboplatin and oxaliplatin.

Forgie and colleagues provided detailed mechanisms of action for these three platinating agents, detailing both the nuclear and cytoplasmatic effects. They also reviewed their current clinical use and limitations, including side effects and mechanisms of resistance [13]. Concerning oxaliplatin, Martinez-Bernabe et al. studied the effect of oxaliplatin on inflammation and cancer stem cell markers in primary and metastatic colorectal tumourspheres, demonstrating the efficiency of this drug in advanced stages of colorectal cancer, but not in the early stages [14].

Several other platinum and nonplatinum metal complexes have shown potent cytotoxic and antitumor properties that are associated with low side effects.

Aiming at reducing the unselective cytotoxicity of cisplatin, inert Pt(IV) prodrugs have been proposed since they can be activated via a reduction by cellular reducing agents or by photoactivation directly in the site of interest. In this context, Canil and colleagues synthesized a new photoactivatable Pt(IV) complex, [Pt(OCOCH_3_)3(4′-phenyl-2,2′:6′,2′′-terpyridine)][CF_3_SO_3_], which is totally unreactive towards selected model biomolecules until its reduction. On the contrary, this compound underwent a rapid and efficient photoreduction with visible light or with flavin, which is naturally present in the cellular environment, leading to its reactive Pt(II) analogue that proved to bind human serum albumin and a monofilament oligonucleotide fragment [15].

Similarly, the research group of Sicilia studied the interaction of a platinum(IV)–salphen complex (Pt(IV)–Sal) with a G-quadruplex, demonstrating the stabilization of the nucleic acid structure by either establishing π-stacking interactions with the terminal G-tetrad or through electrostatic interactions along with H-bond formation [16].

Silconi and coworkers evaluated in vitro and in vivo a previously proposed platinum(IV) complex with alkyl derivatives of thiosalicylic acid, PtCl_2_(*S*-pr-thiosal)_2_. It reduced in vitro the viability of murine B-cell leukaemia lymphoma cells, BCL1, but also decreased the growth of metastases in the leukaemia lymphoma model in BALB/c mice. Moreover, PtCl_2_(*S*-pr-thiosal)_2_ induced apoptosis and cell cycle disturbance, resulting in cell cycle arrest in the G1 phase [17].

Going further with platinum compounds, the research group of Weigand proposed complexes with β-hydroxydithiocinnamic acid esters such as O,S bidendate ligands for Ni(II), Pd (II) and Pt(II) complexes in one paper [18], and Ru(II) and Os(II) complexes in another manuscript [19]. For Ru- and Os-based compounds, the authors demonstrated a selective activity towards ovarian cancer cell lines and cisplatin-resistant cells without showing activity on non-cancerous cells, which were used as the control. Structure–activity relationship (SAR) studies of the Ru(II) compounds suggested that longer alkyl chains at the aromatic ring lead to higher cytotoxic properties. In the osmium complex series, the most active compound proved to be the one with a hydroxy group at the meta-position [19].

In turn, for Ni(II), Pd(II) and Pt(II) complexes, SAR analyses revealed the metal ion (M), alkyl-chain position (P) and length (L) as crucial parameters for an efficient in vitro activity with the following order: M > P > L. The highest activities have been found for some Pd complexes and ortho-substituted compounds [18].

Similarly, Czylkowska et al. proposed a new ligand, i.e., 5-((1-methyl-pyrrol-2-yl) methyl)-4-(naphthalen-1-yl)-1,2,4-triazoline-3-thione, for the development of Mn(II), Fe(II), Ni(II), Cu(II) and Zn(II) complexes. Among these five metal-based complexes, the Mn(II)-based compounds showed the best cytotoxicity against the selected colon and lung cancer cell lines with an activity comparable to that of commercially available drugs, such as 5-fluorouracil and etoposide [20].

Pivovarova and colleagues designed and evaluated copper coordination compounds containing thiazole-based derivatives. In the proposed series, two copper complexes exhibited cytotoxic effects on breast cancer cells with selectivity towards tumourigenic cells over healthy ones, definitively proving the efficacy of the proposed complexation [21].

Finally, in the field of diagnostics, the research group of Shirmanova developed a method for the simultaneous analysis of the cellular metabolic status and oxygen level, using fluorescence lifetime imaging microscopy (FLIM) of metabolic cofactor NAD(P)H, and phosphorescence lifetime imaging (PLIM) of a new polymeric Ir(III)-based sensor (PIr3), respectively [22]. The sensor was derived from a polynorbornene and cyclometalated Ir(III) complex and allowed for the detection of hypoxia, a typical condition of cancer cells, in vitro. Indeed, it resulted in a correlative increase in the PIr3 phosphorescence lifetime and free (glycolytic) NAD(P)H fraction in cells. In vivo, mouse tumours demonstrated a high degree of both metabolic and oxygen states. Both hypoxia and glycolytic metabolism support tumour aggressiveness and resistance to radio-, immuno- and chemotherapies. Therefore, dual FLIM/PLIM metabolic/oxygen imaging will be valuable in preclinical investigations to monitor cancer progression and treatment response [22].

The collection of papers and reviews presented in this Special Issue provides interesting examples of the novel and unique achievements in metal-based compounds with potential anticancer properties.
